# RETRACTION: The effect of negative pressure wound therapy on the outcome of diabetic foot ulcers: A meta‐analysis

**DOI:** 10.1111/iwj.70133

**Published:** 2024-11-12

## Abstract

**RETRACTION:** ZhangN. , LiuY. , YanW. , LiuF. , “The effect of negative pressure wound therapy on the outcome of diabetic foot ulcers: A meta‐analysis,” International Wound Journal 21, no. 4 (2024): e14886, 10.1111/iwj.14886.38651532 PMC11036310

The above article, published online on 23 April 2024, in Wiley Online Library (http://onlinelibrary.wiley.com/), has been retracted by agreement between the journal Editor in Chief, Professor Keith Harding; and John Wiley & Sons, Ltd. Following an investigation by the publisher, the parties have concluded that this article was accepted solely on the basis of a compromised peer review process. Therefore, the article must be retracted. The authors did not respond to the notice of retraction.

**RETRACTION:** N. Zhang , Y. Liu , W. Yan , F. Liu , “The effect of negative pressure wound therapy on the outcome of diabetic foot ulcers: A meta‐analysis,” International Wound Journal 21, no. 4 (2024): e14886, 10.1111/iwj.14886.38651532 PMC11036310

The above article, published online on 23 April 2024, in Wiley Online Library (http://onlinelibrary.wiley.com/), has been retracted by agreement between the journal Editor in Chief, Professor Keith Harding; and John Wiley & Sons, Ltd. Following an investigation by the publisher, the parties have concluded that this article was accepted solely on the basis of a compromised peer review process. Therefore, the article must be retracted. The authors did not respond to the notice of retraction.

